# The Alzheimer's Cell Atlas: A comprehensive brain single‐cell transcriptomic atlas using a generative AI foundation model

**DOI:** 10.1002/alz70855_107196

**Published:** 2025-12-25

**Authors:** Jielin Xu, Yadi Zhou, Noah J Lorincz‐Comi, Lijun Dou, Yuan Hou, Yunguang Qiu, Andrew A. Pieper, Feixiong Cheng

**Affiliations:** ^1^ Cleveland Clinic, Cleveland, OH, USA; ^2^ Case Western Reserve University, Cleveland, OH, USA; ^3^ Louis Stokes Cleveland VA Medical Center, Cleveland, OH, USA; ^4^ Brain Health Medicines Center, Harrington Discovery Institute, University Hospitals Cleveland Medical Center, Cleveland, OH, USA; ^5^ Cleveland Clinic Genome Center, Cleveland, OH, USA

## Abstract

**Background:**

Studying Alzheimer's Disease (AD) at the single‐cell level is essential for uncovering its complex biology, such as identifying disease‐associated cell states and decoding their molecular mechanisms, also even designing personalized treatment strategies.

**Method:**

In this study, we created a human brain single‐nuclei RNA‐seq (snRNA‐seq) atlas focused on neurological disorders. In addition to standard quality controls, we conducted meta clinical data harmonization which combined both pathological and clinical diagnoses to define disease groups. We performed snRNA‐seq data harmonization and cell type annotation using a generative AI‐based single‐cell foundation model. The snRNA‐seq data harmonization accounted for both categorical and continuous covariates, and cell type annotation was performed hierarchically using both supervised and un‐supervised learnings.

**Result:**

Our human brain snRNA‐seq atlas comprised approximately 14 million nuclei—making it, to the best of our knowledge, the largest human Alzheimer's brain‐cell atlas of its kind. After the metadata harmonization, our atlas included 2,239 human postmortem samples, including 1031 control, 658 AD, 110 AD‐resilience, 221 primary age‐related tauopathy (PART) samples, and 219 samples with other neurological diseases. The atlas covers 33 brain regions, broad age ranges from 19 to 100+, and various clinical factors such as *APOE*genotype, Braak stage, clinical and pathological diagnoses. We identified over 50 cell types at the finest level of annotation (3rd level), such as astrocyte, T cell, microglia subtypes e.g., DAM, tau, and MHC‐microglia, excitatory neuron subtypes, such as L2/3‐intratelencephalic (IT), inhibitory neuron subtypes, e.g., Sst, also other neuron subtypes from non‐cortex regions, such as dopaminergic neuron. We found loss of Sst neuron has been consistently observed in AD compared with resilience samples. Our DEG analyses revealed proteins potentially linked to cognitive resilience in AD, e.g., HSP90AA1, CLU in Sst neuron, PLCG2, TSPAN14 in DAM, and ERCC1 in T cells. Additional analyses revealed decreased PLCG2 expression in DAM with aging in AD subjects, but not in cognitively healthy individuals, indicating potential role of PLCG2 in resilience.

**Conclusion:**

This study generated a large‐scale human brain snRNA‐seq atlas focused on neurodegenerative diseases, providing a valuable digital resource for the scientific community to explore multiple neurodegenerative conditions from diverse perspectives.

